# A retrospective analysis of pembrolizumab plus chemotherapy versus pembrolizumab monotherapy for advanced or recurrent non‐small cell lung cancer

**DOI:** 10.1111/1759-7714.13915

**Published:** 2021-03-12

**Authors:** Taisuke Isono, Naho Kagiyama, Shun Shibata, Hitomi Nakajima, Yuma Matsui, Kenji Takano, Takashi Nishida, Chiaki Hosoda, Eriko Kawate, Yoichi Kobayashi, Takashi Ishiguro, Yotaro Takaku, Kazuyoshi Kurashima, Tsutomu Yanagisawa, Noboru Takayanagi

**Affiliations:** ^1^ Department of Respiratory Medicine Saitama Cardiovascular and Respiratory Center Saitama Japan

**Keywords:** adverse event, combination therapy, immune checkpoint inhibitor, overall survival, treatment discontinuation

## Abstract

**Background:**

Although clinical trials have investigated the addition of pembrolizumab to chemotherapy for non‐small cell lung cancer, none have investigated the addition of chemotherapy to pembrolizumab.

**Methods:**

We conducted a retrospective study of 71 NSCLC patients including 33 treated with pembrolizumab plus chemotherapy (combination therapy group) and 38 treated with pembrolizumab monotherapy (monotherapy group) from 1 May 2016 to 31 August 2020.

**Results:**

Eleven of 33 (33.3%) patients in the combination therapy group and 37 of 38 (97.4%) patients in the monotherapy group had programmed cell death ligand‐1 (PD‐L1) tumor proportion score (TPS) ≥50%. Objective response rate (ORR) and median overall survival (OS) were not significantly different between the combination therapy group and monotherapy group (54.5% vs. 47.4, *p* = 0.637 and 16.6 vs. 27.0 months, *p* = 0.463). In patients with PD‐L1 TPS ≥50%, ORR and median OS were not different between the combination therapy group and the monotherapy group (63.6% vs. 48.6%, *p* = 0.499 and not reached vs. 27.0 months, *p* = 0.976). Thirty‐three (100%) patients experienced adverse events (AEs) in the combination therapy group and 32 (84.2%) in the monotherapy group. Treatment discontinuation at 1 year due to AEs occurred more frequently in the combination therapy group (45.2%) than in the monotherapy group (21.1%).

**Conclusion:**

There was no significant difference in ORR and OS between the two groups, and treatment discontinuation was more frequent in the combination group. A randomized controlled trial is needed to evaluate the addition of chemotherapy to pembrolizumab for first‐line treatment in patients with PD‐L1 TPS ≥50%.

## Graphical abstract

There was no significant difference in clinical response and prognosis between the combination therapy group and the monotherapy group. We think that the higher frequency of treatment discontinuation due to adverse events in the combination therapy group compared to the monotherapy group is one reason why there was no significant difference in prognosis between the two groups.

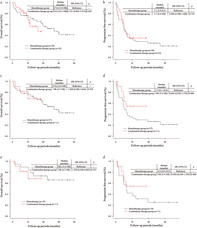

## INTRODUCTION

Immune checkpoint inhibitors (ICIs) have become a standard treatment in advanced non‐small cell lung cancer (NSCLC) patients. In phase 3 trials, NSCLC patients treated with ICI monotherapies had longer median overall survival (OS) than those treated with docetaxel in second‐line treatment.^1^, ^2^, ^3^, ^4^ Moreover, the KEYNOTE‐024 and KEYNOTE‐042 trials revealed that NSCLC patients with programmed cell death ligand‐1(PD‐L1) tumor proportion score (TPS) ≥50% who were treated with first‐line pembrolizumab monotherapy had a longer median OS than those treated with first‐line platinum‐based chemotherapy.^5^, ^6^

Recently, phase 3 trials showed that NSCLC patients treated with ICI plus platinum‐based chemotherapy as first‐line treatment had a higher objective response rate (ORR), longer median progression‐free survival (PFS), and longer median OS than those treated with chemotherapy alone, regardless of the PD‐L1 TPS.^7^, ^8^, ^9^, ^10^, ^11^ Therefore, both ICIs plus chemotherapy and pembrolizumab monotherapy are available for NSCLC patients with PD‐L1 TPS ≥50%. However, no clinical trials have compared ICIs plus chemotherapy to pembrolizumab monotherapy.

Additionally, patients with an Eastern Cooperative Oncology Group performance status (ECOG PS) of ≥2 were excluded, and the number of patients aged ≥75 years was small in phase 3 trials. No reports evaluated first‐line pembrolizumab plus chemotherapy for patients with ECOG PS of ≥2 and patients aged ≥75 years in the clinical setting. A few reports evaluated first‐line pembrolizumab monotherapy in NSCLC patients in the clinical setting and showed that adverse events (AEs) occurred more frequently in patients aged ≥75 years and poor clinical response in patients with poor ECOG PS.^12^, ^13^, ^14^, ^15^

We therefore retrospectively reviewed the clinical data of NSCLC patients treated with pembrolizumab plus chemotherapy and patients treated with pembrolizumab monotherapy as first‐line treatment and aimed to compare efficacy and safety between them and investigate the efficacy and safety in patients with ECOG PS of ≥2 and patients aged ≥75 years.

## METHODS

### Subjects

From 1 May 2016 to 31 March 2020, 71 patients with unresectable advanced or recurrent NSCLC were treated with pembrolizumab plus chemotherapy (combination therapy group) or pembrolizumab monotherapy (monotherapy group) as first‐line treatment at the Saitama Cardiovascular and Respiratory Center. The diagnosis of lung cancer was based on pathology or cytology findings. The clinical stage was established according to the 8th edition of the TNM classification. Information concerning tumorous characteristics including epidermal growth factor receptor (EGFR) mutation, anaplastic lymphoma kinase (ALK) rearrangement, c‐ros oncogene 1 (ROS‐1) rearrangement, BRAF V600E mutation, and PD‐L1 TPS was collected. The PD‐L1 TPS was assessed by means of the PD‐L1 immunohistochemistry 22C3 pharmDx assay. Treatments were administered until disease progression, intolerable toxicity, or patient refusal occurred. Treatment discontinuation was defined as discontinuation of any treatment, including pembrolizumab and/or chemotherapy.

### Study design

We retrospectively investigated patient background, ORR, OS, and development of AEs, including immune‐related AEs (irAEs). We also investigated the predictive factors for OS. Clinical data were collected from medical records. Baseline clinical parameters were obtained within 1 month of the initial diagnosis. Tumor evaluation was assessed according to Response Evaluation Criteria in Solid Tumors (RECIST) version 1.1.^16^ ORR was defined as the proportion of patients achieving complete response or partial response. OS was measured from first administration of the first‐line treatment to death. The data cut‐off date was 31 August 2020. The AEs were assessed using National Cancer Institute Common Terminology Criteria for Adverse Events (CTCAE) version 4.0. This study was conducted in accordance with the Declaration of Helsinki and was approved by the Institutional Review Board of the Saitama Cardiovascular and Respiratory Center, which waived the necessity to obtain informed consent.

### Statistical analysis

Categorical data are summarized by frequency and percent, and continuous data are reported as the median and range. Statistical analyses were performed by using Fisher's test for binary data and the Mann–Whitney U test for continuous data. The Kaplan–Meier method was used to estimate median OS. Cumulative incidence of discontinuation of treatment due to AEs was estimated using Gray's test with discontinuation of treatment due to disease progression or death as the competing event. A *p* value of <0.05 was considered statistically significant. The factors with *p* value of <0.05 on univariate analysis were included in the multivariate analysis. All statistical analyses were performed with EZR version 1.36 (Saitama Medical Center, Jichi Medical University), which is a graphical user interface for R (The R Foundation for Statistical Computing, version 3.4.3).^17^

## RESULTS

### Patient characteristics

Of the 71 patients with advanced NSCLC who underwent first‐line pembrolizumab therapy, 33 were included in the combination therapy group and 38 were included in the monotherapy group. The baseline patient characteristics of the two groups are summarized in Table 1. The median age and percentage of patients aged ≥75 years were significantly higher in the monotherapy group versus the combination group (72 vs. 66 years and 36.8% vs. 6.1%, respectively). Patients with an ECOG PS of 2 or 3 were present only in the monotherapy group. Other than one patient with ROS1 fusion in the monotherapy group, no patients had an EGFR mutation, ALK fusion, or a BRAF mutation in either group. In the combination therapy group, the numbers of patients with PD‐L1 TPS ≥50%, 1–49%, and <1% were 11 (33.3%), seven (21.2%), and 10 (30.3%), respectively. All but one patient in the monotherapy group had PD‐L1 TPS ≥50%. The median follow‐up period was significantly shorter in the combination therapy group versus monotherapy group (9.7 vs. 12.0 months).

**TABLE 1:** Characteristics of patients and objective response rate in both groups

	Combination therapy group (*n* = 33)	Monotherapy group (*n* = 38)	*p* value
Chemotherapy regimen added to pembrolizumab			
CDDP + PEM	7 (21.2)	−	−
CDBCA + PEM	16 (45.8)	−	−
CDBCA + nab‐PTX	10 (20.3)	−	−
Age at first‐line therapy initiation (years)	66 (44–77)	72 (57–82)	<0.001
Patients aged ≥75 years	2 (6.1)	14 (36.8)	0.003
Sex, male	31 (93.9)	28 (73.7)	0.029
Smoker	30 (90.9)	33 (86.8)	0.716
ECOG PS			0.027
0 or 1	33 (100.0)	32 (84.2)	
2 or 3	0 (0.0)	6 (15.8)	
Stage			0.775
III	7 (21.2)	7 (18.4)	
IV	26 (78.8)	31 (81.6)	
Pre‐existing respiratory disease			
Emphysema	16 (48.5)	21 (55.3)	0.638
IIPs	4 (12.1)	4 (10.5)	1.000
Radiation‐induced pulmonary fibrosis	0 (0.0)	1 (2.6)	1.000
Asthma	1 (3.0)	3 (7.9)	0.618
Autoimmune disease			
Chronic thyroiditis	0 (0.0)	1 (2.6)	1.000
Basedow's disease	0 (0.0)	1 (2.6)	1.000
Use of corticosteroid or immunosuppressant	4 (12.1)	2 (5.3)	0.406
Histologic type			0.776
Adenocarcinoma	20 (60.6)	22 (57.9)	
Squamous cell carcinoma	7 (21.2)	8 (21.1)	
Pleomorphic carcinoma	0 (0.0)	2 (5.3)	
LCNEC	1 (3.0)	0 (0.0)	
NOS	5 (15.2)	6 (15.8)	
EGFR mutation			1.000
+	0 (0.0)	0 (0.0)	
−	29 (87.9)	35 (92.1)	
NA	4 (12.1)	3 (7.9)	
ALK rearrangement			1.000
+	0 (0.0)	0 (0.0)	
−	27 (81.8)	33 (86.8)	
NA	6 (18.2)	5 (13.2)	
ROS‐1 rearrangement			1.000
+	0 (0.0)	0 (0.0)	
−	27 (81.8)	26 (68.4)	
NA	6 (18.2)	11 (28.9)	
BRAF V600E mutation			1.000
+	0 (0.0)	0 (0.0)	
−	20 (60.6)	8 (21.1)	
NA	13 (39.4)	30 (78.9)	
PD‐L1 TPS			<0.001
≥50%	11 (33.3)	37 (97.4)	
1–49%	7 (21.2)	1 (2.6)	
<1%	10 (30.3)	0 (0.0)	
NA	5 (15.2)	0 (0.0)	
Brain metastasis	6 (18.2)	8 (21.1)	1.000
Prior treatment for brain metastasis	4 (12.1)	6 (15.8)	1.000
Prior radiotherapy	4 (12.1)	5 (13.2)	1.000
Prior thoracic radiotherapy	1 (3.0)	2 (5.3)	1.000
Follow‐up period (months)	9.7 (1.8–18.9)	12.0 (0.4–40.3)	0.002
Best overall response			
CR	0 (0.0)	0 (0.0)	
PR	18 (54.5)	18 (47.4)	
SD	9 (27.3)	8 (21.1)	
PD	6 (18.2)	12 (31.6)	
**ORR**	54.5 (18/33)	47.4 (18/38)	0.637
ORR by PD‐L1 TPS			
≥50%	63.6 (7/11)	48.6 (18/37)	0.499
1–49%	57.1 (4/7)	0.0 (0/1)	1.000
<1%	30.0 (3/10)	NA	NA
NA	80.0 (4/5)	NA	NA
ORR in patients aged < 75 years with PD‐L1 TPS ≥ 50% and ECOG PS of 0 or 1	63.6 (7/11)	63.2 (12/19)	1.000

*Note*: Data are presented as *n*, median (range) or *n* (%). ORRs are presented as % (response/overall).

*Abbreviations*: ALK, anaplastic lymphoma kinase; CBDCA, carboplatin; CDDP, cisplatin; CR, complete response; EGFR, epidermal growth factor receptor; IIPs, idiopathic interstitial pneumonias; LCNEC, large‐cell neuroendocrine carcinoma; NA, not available; nab‐PTX, nanoparticle albumin‐bound paclitaxel; NOS, not otherwise specified; ORR, objective response rate; PD, progressive disease; PD‐L1, programmed cell death ligand‐1; ECOG PS, Eastern Cooperative Oncology Group performance status; PEM, pemetrexed; PR, partial response; ROS‐1, c‐ros oncogene 1; SD, stable disease; TPS, tumor proportion score.

### Efficacy

In the combination therapy group and the monotherapy group, partial response (PR) was achieved in 18 (54.5%) and 18 (47.4%) of patients, stable disease (SD) was present in nine (27.3%) and eight (21.1%) patients, and progressive disease (PD) was present in six (18.2%) and 12 (31.6%) patients, respectively (Table 1). There was no significant difference in the ORR between patients in the combination therapy group versus the monotherapy group (54.4% vs. 47.4%, odds ratio [OR] 1.328, 95% confidence interval [CI] 0.473–3.768, *p* = 0.637) or in patients with PD‐L1 TPS ≥50% (63.6% vs. 48.6%, OR 1.824, 95% CI 0.384–10.016, *p* = 0.499). Furthermore, in patients aged <75 years with PD‐L1 TPS ≥50% and ECOG PS of 0 or 1, the difference in ORR between the combination therapy group and the monotherapy group was not significant (63.6% vs. 63.2%, OR 0.980, 95% CI 0.152–5.761, *p* = 1.000).

**FIGURE 1:**
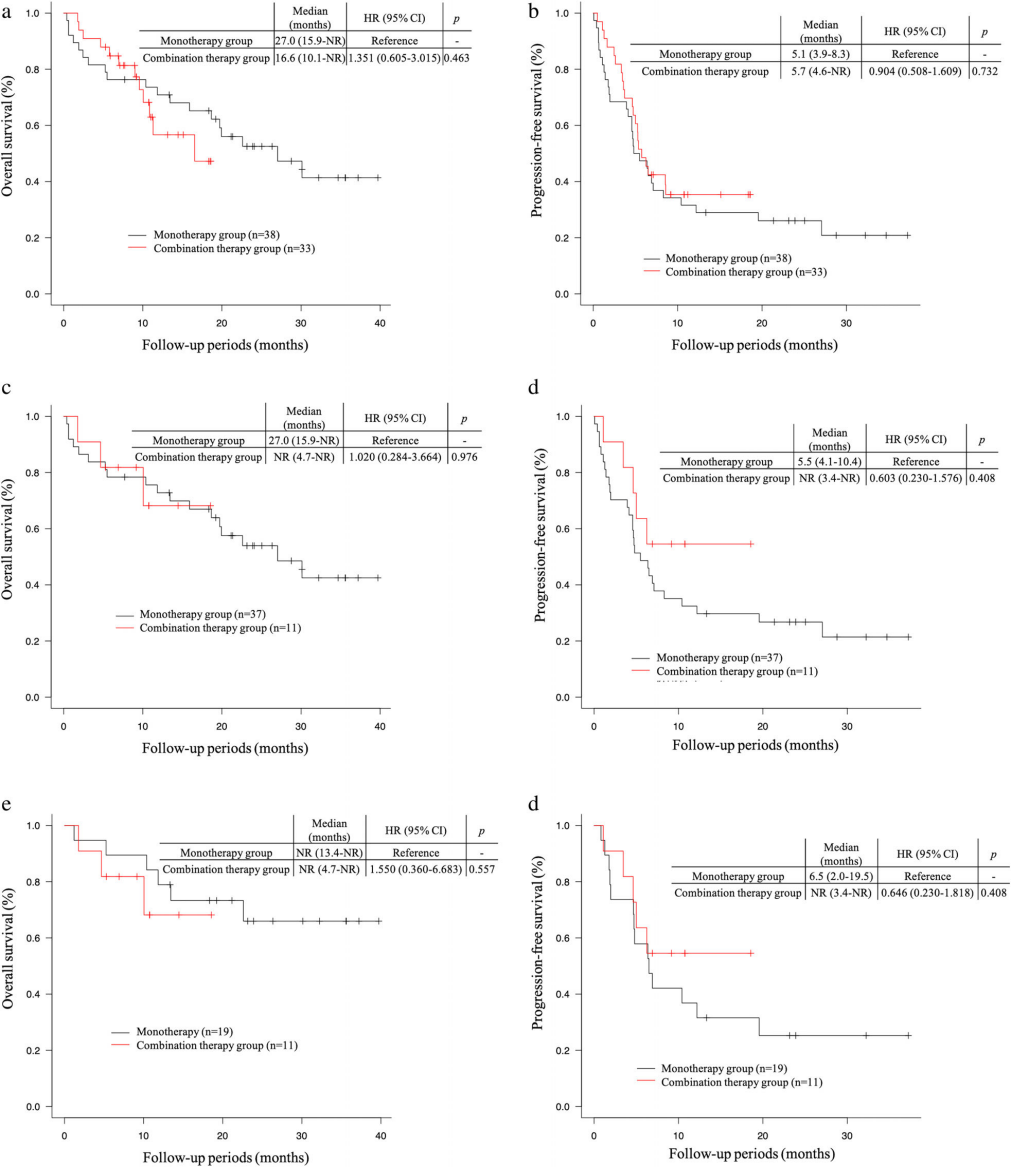
Kaplan–Meier curves showing (a) OS in both groups, (b) OS stratified by PD‐L1 TPS in the combination therapy group, (c) OS in patients with PD‐L1 TPS ≥50% in both groups, and (d) OS in patients aged <75 years with PD‐L1 TPS ≥50% and ECOG PS of 0 or 1 in both groups. CI, confidence interval; ECOG PS, Eastern Cooperative Oncology Group performance status; HR, hazard ratio; NA, not available; NR, not reached; OS, overall survival; PD‐L1, programmed cell death ligand‐1; TPS, tumor proportion score

The median OS was 16.6 months (95% CI 10.1–not reached [NR]) in the combination therapy group and 27.0 months (95% CI 15.9–NR) in the monotherapy group (hazard ratio [HR] for death 1.351, 95% CI 0.605–3.015, *p* = 0.463) (Figure 1(a)). The median PFS was 5.7 months (95% CI 4.6–NR) in the combination therapy group and 5.1 months (95% CI 3.9–8.3) in the monotherapy group (HR for death 1.351, 95% CI 0.605–3.015, *p* = 0.463) (Figure 1(b)). In patients with PD‐L1 TPS ≥50%, the median OS was NR (95% CI 4.7–NR) in the combination therapy group and 27.0 months (95% CI 15.9–NR) in the monotherapy group (HR for death 1.020, 95% CI 0.284–3.664, *p* = 0.976), and the median PFS was NR (95% CI 3.4–NR) in the combination therapy group and 5.5 months (95% CI 4.1–10.4) in the monotherapy group (HR for death 0.603, 95% CI 0.230–1.576, *p* = 0.408) (Figure 1(c),(d)). Moreover, in patients aged <75 years with PD‐L1 TPS ≥50% and ECOG PS of 0 or 1, the median OS was NR (95% CI 4.7–NR) in the combination therapy group and NR (95% CI 13.4–NR) in the monotherapy group (HR for death 1.550, 95% CI 0.360–6.683, *p* = 0.557), and the median PFS was NR (95% CI 3.4–NR) in the combination therapy group and 6.5 months (95% CI 2.0–19.5) in the monotherapy group (HR for death 0.646, 95% CI 0.230–1.818, *p* = 0.408) (Figure 1(e),(f)).

### Safety

AEs occurred in 33 patients (100%) in the combination therapy group and 32 (84.2%) in the monotherapy group (Table 2). Fifteen (45.5%) patients in the combination therapy group and 17 (44.7%) in the monotherapy group experienced AEs of grade ≥3. Total AEs of grade ≥3 totaled 39 in the combination therapy group and 20 in the monotherapy group, therefore patients who developed multiple AEs of grade ≥3 were more frequent in the combination therapy group. Anorexia or nausea, neutropenia, anemia, and thrombocytopenia occurred more frequently in the combination therapy group than in the monotherapy group. irAEs developed in 32 patients (97.0%) in the combination therapy group and in 28 (73.7%) patients in the monotherapy group. In patients with PD‐L1 TPS ≥50%, irAEs developed in 11 patients (100.0%) in the combination therapy group and 30 (81.1%) patients in the monotherapy group (Supporting Information Table SS1). Six (18.2%) patients experienced irAEs of grade ≥3 in the combination therapy group, as did six (15.8%) in the monotherapy group. Liver injury, renal dysfunction, and colitis or diarrhea occurred more frequently in the combination therapy group than in the monotherapy group. AEs of grade 5 were septic shock in one patient in the combination therapy group and pneumonitis in one patient in the monotherapy group. Treatment was discontinued because of AEs in 14 patients (42.4%) in the combination therapy group and nine (23.7%) patients in the monotherapy group during the overall observation period. In patients with PD‐L1 TPS ≥50%, treatment was discontinued because of AEs in five patients (45.5%) in the combination therapy group and nine (24.3%) patients in the monotherapy group during the overall observation period.

**TABLE 2:** Adverse events including immune‐related adverse events

Events	Combination therapy group (*n* = 33)			Monotherapy group (*n* = 38)		
	All	Grade ≥3	Discontinuation	All	Grade ≥3	Discontinuation
Any AEs including irAEs	33 (100.0)	15 (45.5)	14 (42.4)	32 (84.2)	17 (44.7)	9 (23.7)
Anorexia or nausea	22 (66.7)	1 (3.0)	0 (0.0)	0 (0.0)	NA	NA
Neutropenia	19 (57.6)	8 (24.2)	0 (0.0)	1 (2.6)	0 (0.0)	0 (0.0)
Anemia	14 (42.4)	4 (12.1)	0 (0.0)	1 (2.6)	1 (2.6)	0 (0.0)
Thrombocytopenia	13 (39.4)	3 (9.1)	0 (0.0)	1 (2.6)	0 (0.0)	0 (0.0)
Drug‐related fever	5 (15.2)	0 (0.0)	0 (0.0)	7 (18.4)	0 (0.0)	0 (0.0)
Pneumonia	5 (15.2)	4 (12.1)	1 (3.0)	3 (7.9)	1 (2.6)	0 (0.0)
Hiccups	4 (12.1)	0 (0.0)	0 (0.0)	0 (0.0)	NA	NA
Febrile neutropenia	2 (6.1)	2 (6.1)	0 (0.0)	0 (0.0)	NA	NA
Herpes zoster	2 (6.1)	0 (0.0)	0 (0.0)	0 (0.0)	NA	NA
PTE	1 (3.0)	1 (3.0)	1 (3.0)	0 (0.0)	NA	NA
DVT	1 (3.0)	0 (0.0)	0 (0.0)	0 (0.0)	NA	NA
Septic shock	1 (3.0)	1 (3.0)	1 (3.0)	0 (0.0)	NA	NA
Asthma	0 (0.0)	NA	0 (0.0)	2 (5.3)	1 (2.6)	0 (0.0)
Any irAEs	32 (97.0)	6 (18.2)	11 (33.3)	28 (73.7)	6 (15.8)	9 (23.7)
Hepatitis	21 (63.6)	2 (6.1)	2 (6.1)	14 (36.8)	1 (2.6)	1 (2.6)
Rash	12 (36.4)	3 (9.1%	3 (9.1)	14 (36.8)	0 (0.0)	1 (2.6)
Nephritis	12 (36.4)	1 (3.0%	6 (18.2)	8 (21.1)	0 (0.0)	0 (0.0)
Colitis or diarrhea	8 (24.2)	0 (0.0)	0 (0.0)	2 (5.3)	0 (0.0)	0 (0.0)
Pneumonitis	4 (12.1)	0 (0.0)	4 (12.1)	7 (18.4)	3 (7.9)	6 (15.8)
Thyroid dysfunction	4 (12.1)	0 (0.0)	0 (0.0)	5 (13.2)	0 (0.0)	0 (0.0)
Hypoparathyroidism	1 (3.0)	0 (0.0)	0 (0.0)	0 (0.0)	NA	NA
Isolated ACTH deficiency	0 (0.0)	NA	NA	1 (2.6)	1 (2.6)	0 (0.0)
Arthritis	0 (0.0)	NA	NA	1 (2.6)	0 (0.0)	0 (0.0)
Eosinophilic fasciitis	0 (0.0)	NA	NA	1 (2.6)	1 (2.6)	1 (2.6)
**Total AEs**	122	39		47	20	

*Note*: Data are presented as *n*, median (range) or *n* (%).

*Abbreviations*: ACTH, adrenocorticotropic hormone; AEs, adverse events; DVT, deep venous thrombosis; irAEs, immune‐related adverse events; NA, not available; PTE, pulmonary thromboembolism.

### Continuation of treatment

The continuation of treatment at the end of the observation period, 3 months, 6 months, and 1 year is summarized in Table 3. Treatment discontinuation due to AEs occurred more frequently in the combination therapy group than in the monotherapy group, but treatment discontinuation due to PD occurred more frequently in the monotherapy group than in the combination therapy group at the end of the observation period. However, it was necessary to compare the two groups in separate observation periods because the follow‐up period was shorter in the combination therapy group than that in the monotherapy group. In the combination therapy group, only one patient was able to continue treatment at 1 year, and treatment discontinuation due to AEs occurred more frequently than in the monotherapy group at 3 months (24.2% vs. 13.2%), 6 months (36.4% vs. 15.8%), and 1 year (45.2% vs. 21.1%). In patients with PD‐L1 TPS ≥50%, treatment discontinuation due to AEs occurred more frequently in the combination therapy group (Supporting Information Table S2).

**TABLE 3:** Continuation of first‐line treatment

	End of observation period	At 3 months	At 6 months	At 1 year
Combination therapy group				
n	33	33	33	31
Continuation of first‐line treatment	3 (9.1)	16 (48.5)	6 (18.2)	1 (3.2)
Discontinuation due to PD	16 (48.5)	9 (27.3)	15 (45.5)	16 (51.6)
Discontinuation due to AEs	14 (42.4)	8 (24.2)	12 (36.4)	14 (45.2)
Discontinuation due to irAEs	11 (33.3)	5 (15.2)	9 (27.3)	11 (35.5)
Discontinuation due to others	0 (0.0)	0 (0.0)	0 (0.0)	0 (0.0)
Monotherapy group				
n	38	38	38	38
Continuation of first‐line treatment	6 (15.8)	21 (55.3)	13 (34.2)	7 (18.4)
Discontinuation due to PD	22 (57.9)	12 (31.6)	19 (50.0)	22 (57.9)
Discontinuation due to AEs	9 (23.7)	5 (13.2)	6 (15.8)	8 (21.1)
Discontinuation due to irAEs	9 (23.7)	5 (13.2)	6 (15.8)	8 (21.1)
Discontinuation due to others	1 (2.6)	0 (0.0)	0 (0.0)	1 (2.6)

*Note*: Data are presented as *n* (%).

*Abbreviations*: AEs, adverse events; irAEs, immune‐related adverse events; PD, progressive disease.

**FIGURE 2:**
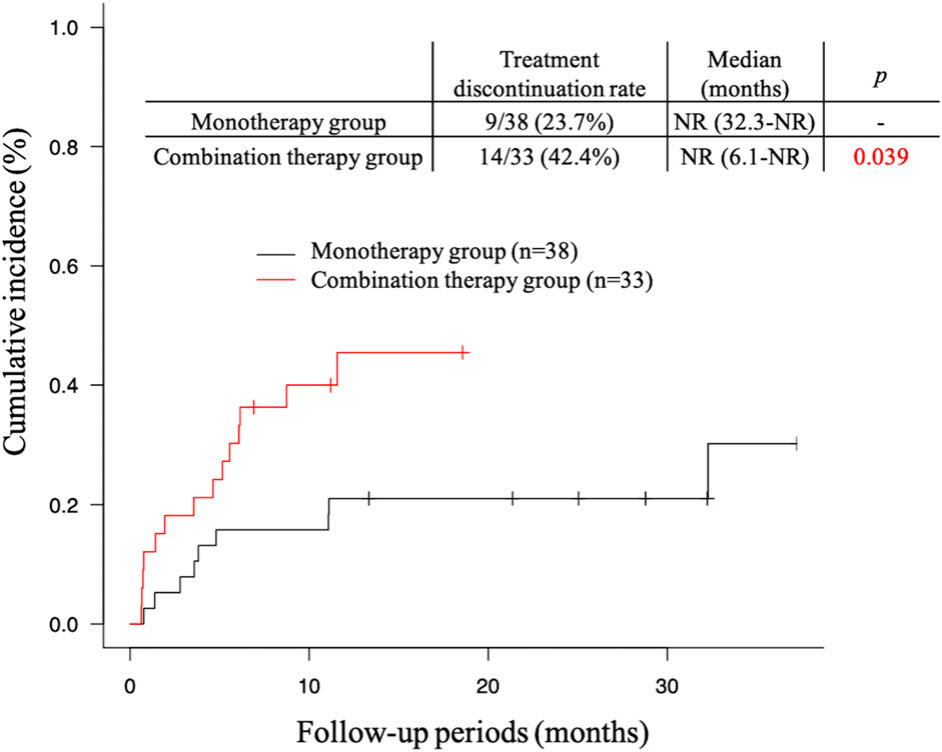
Cumulative incidence of treatment discontinuation due to adverse events in the combination therapy group and in the monotherapy group. Treatment was discontinued significantly more frequently and earlier in the combination therapy group than in the monotherapy group. NR, not reached

The causes of treatment discontinuation due to AEs at 1 year are summarized in Table 4. Treatment discontinuation was observed more frequently in the combination therapy group (35.5%) than in the monotherapy group (21.1%). Furthermore, treatment discontinuation at 1 year due to AEs other than irAEs was observed in only three patients (9.7%) in the combination therapy group. Of the 14 patients discontinuing treatment due to AEs in the combination therapy group, eight (25.8%) discontinued pembrolizumab, including four discontinuing only pembrolizumab and four discontinuing all treatment, and six (19.4%) discontinued only chemotherapy. The cumulative incidence of treatment discontinuation due to AEs (Figure 2) was significantly higher in the combination therapy group than in the monotherapy group (*p* = 0.039). Cumulative incidence curves showed the onset of treatment discontinuation due to AEs occurred earlier in the combination therapy group than in the monotherapy group.

**TABLE 4:** Reasons for treatment discontinuation at 1 year

	Combination therapy group (*n* = 31)	Monotherapy group (*n* = 38)
Any AEs	14 (45.2)	8 (21.1)
AEs other than irAEs	3 (9.7)	0 (0.0)
Pneumonia	1 (3.2)	
Pulmonary thromboembolism	1 (3.2)	
Septic shock	1 (3.2)	
irAEs		
Nephritis	6 (19.4)	
Pneumonitis	3 (9.7)	6 (15.8)
Hepatitis	2 (6.5)	1 (2.6)
Eosinophilic fasciitis		1 (2.6)

*Note*: Data are presented as *n* (%).

*Abbreviations*: AEs, adverse events; irAEs, immune‐related adverse events.

### Efficacy and safety of pembrolizumab in elderly patients and those with poor ECOG PS

Two patients in the combination therapy group and 14 in the monotherapy group were aged ≥75 years (Table 5). Response was achieved in one of two patients (50.0%) in the combination therapy group and in six of 14 patients (42.9%) in the monotherapy group. One (50.0%) patient in the combination therapy group and three (21.4%) in the monotherapy group experienced AEs of grade ≥3.

**TABLE 5:** Patients aged ≥75 years and patients with ECOG PS of 2 or 3

No.	Age/sex	Regimen	ECOG PS	Pre‐existing respiratory disease	Pathology	PD‐L1 TPS	Best overall response	OS (months)	AEs of grade ≥3
Patients aged ≥ 75 years									
1	75/male	Pembrolizumab	0	None	Adenocarcinoma	≥50%	PR	27.4	Pneumonia
2	75/male	Pembrolizumab	0	Emphysema	Adenocarcinoma	≥50%	PR	29.2	–
3	76/male	Pembrolizumab	1	None	Adenocarcinoma	≥50%	PD	1.9	–
4	76/male	Pembrolizumab	1	Emphysema	Adenocarcinoma	≥50%	PR	35.2	–
5	76/male	Pembrolizumab	1	Emphysema	Pleomorphic carcinoma	≥50%	PR	21.7	–
6	76/male	Pembrolizumab	0	Emphysema	Adenocarcinoma	≥50%	SD	20.0	–
7	77/male	Pembrolizumab	1	Emphysema	Squamous cell carcinoma	≥50%	PR	24.4	–
8	77/male	Pembrolizumab	0	Emphysema	NOS	≥50%	SD	16.1	–
9	78/female	Pembrolizumab	1	Emphysema	Squamous cell carcinoma	≥50%	SD	20.2	–
10	79/male	Pembrolizumab	1	None	Adenocarcinoma	≥50%	PD	7.8	–
11	81/female	Pembrolizumab	1	None	Adenocarcinoma	≥50%	PR	5.6	–
12	81/female	Pembrolizumab	1	Emphysema	Squamous cell carcinoma	≥50%	SD	18.9	Anemia
13	82/male	Pembrolizumab	3	Emphysema	NOS	≥50%	PD	0.6	–
14	82/female	Pembrolizumab	1	Asthma	Adenocarcinoma	1–49%	PD	2.5	Asthma
15	75/female	CBDCA+PEM+ Pembrolizumab	1	None	NOS	0%	SD	6.0	Neutropenia
16	77/male	CBDCA+PEM+ Pembrolizumab	1	IIPs	Adenocarcinoma	1–49%	PR	7.1	–
Patients with ECOG PS of 2 or 3									
1	70/male	Pembrolizumab	2	IIPs	Adenocarcinoma	≥50%	PD	0.4	Pneumonitis
2	63/female	Pembrolizumab	2	None	Adenocarcinoma	≥50%	PD	3.2	–
3	74/male	Pembrolizumab	2	None	Adenocarcinoma	≥50%	PD	0.6	–
4	67/male	Pembrolizumab	2	Emphysema	Adenocarcinoma	≥50%	SD	25.4	–
5	66/male	Pembrolizumab	3	Emphysema	Squamous cell carcinoma	≥50%	PR	30.6	–
6	82/male	Pembrolizumab	3	Emphysema	NOS	≥50%	PD	0.6	–

*Abbreviations*: AEs, adverse events; CBDCA, carboplatin; ECOG PS, Eastern Cooperative Oncology Group performance status; IIPs, idiopathic interstitial pneumonias; NOS, not otherwise specified; OS, overall survival; PD, progressive disease; PD‐L1, programmed cell death ligand‐1; PEM, pemetrexed; PR, partial response; SD, stable disease; TPS, tumor proportion score.

No patient had an ECOG PS of 2 or 3 in the combination group, whereas six patients had this score in the monotherapy group (Table 5). Response was obtained in one of these six patients (16.7%). Cases 4 and 5 survived for more than 2 years. One (16.7%) patient experienced AEs of grade ≥3.

## DISCUSSION

This study revealed that the addition of chemotherapy to pembrolizumab did not contribute to improvement of clinical response or prognosis in patients overall, in patients with PD‐L1 ≥50%, or in patients aged <75 years with PD‐L1 TPS ≥50% and ECOG PS of 0 or 1. AEs developed more frequently in the combination therapy group than in the monotherapy group. Treatment discontinuation due to AEs occurred more frequently and earlier in the combination therapy group. The response and frequency of AEs of grade ≥3 in patients aged ≥75 years were similar to those in the patients overall. Patients with poor ECOG PS had a poor response to pembrolizumab.

The efficacy of pembrolizumab plus chemotherapy and pembrolizumab monotherapy as first‐line treatment in NSCLC was previously reported. The phase 3 KEYNOTE‐189 trial enrolled non‐squamous NSCLC patients, and the phase 3 KEYNOTE‐407 trial enrolled squamous NSCLC patients for evaluation of first‐line pembrolizumab plus chemotherapy.^7^, ^8^ In the KEYNOTE‐189 trial, the ORR was 48.3%, median OS was 22.0 months, and subgroup analysis of patients with PD‐L1 TPS ≥50% showed an ORR of 62.1% and median OS of 27.7 months in patients treated with pembrolizumab plus chemotherapy. In the KEYNOTE‐407 trial, the ORR was 62.6%, median OS was 17.1 months, and subgroup analysis of patients with PD‐L1 TPS ≥1% showed an ORR of 59.1% and median OS of 18.9 months in patients treated with pembrolizumab plus chemotherapy. The phase 3 KEYNOTE‐024 trial enrolled patients with PD‐L1 TPS ≥50%, and the phase 3 KEYNOTE‐042 trial enrolled patients with PD‐L1 TPS ≥1% to evaluate first‐line pembrolizumab monotherapy.^5^, ^6^ In the KEYNOTE‐024 trial, the ORR was 45.5% and median OS was 30.0 months in patients treated with pembrolizumab monotherapy. In the KEYNOTE‐042 trial, the ORR was 27%, median OS was 16.9 months, and subgroup analysis of the patients with PD‐L1 TPS ≥50% showed an ORR of 39% and median OS of 20.0 months in patients treated with pembrolizumab monotherapy.

In contrast, several studies reported the efficacy of first‐line pembrolizumab monotherapy for NSCLC patients with PD‐L1 TPS ≥50% in the clinical setting. Tambo et al. studied 95 patients and showed an ORR of 40.0% and OS of NR.^18^ Amrane et al. studied 108 patients and showed that the ORR was 57.3% and median OS was 15.2 months.^19^ Aguilar et al. investigated 187 patients and reported an ORR of 44.4% and median OS of NR.^20^ Tamiya et al. studied 213 patients and found an ORR of 51.2% and median OS of 17.8 months.^21^ Cortellini et al. studied 1010 patients and reported an ORR of 48.9% and median OS of 27.4 months.^22^ No reports evaluated the efficacy of pembrolizumab plus chemotherapy in the clinical setting or compared the efficacy of pembrolizumab plus chemotherapy and pembrolizumab monotherapy. The present study revealed that the ORR and median OS in both groups were similar to those of the previous clinical trials and retrospective studies, and there were no significant differences in the ORR and median OS between the two groups in the clinical setting.

Several studies evaluated the safety of pembrolizumab plus chemotherapy and pembrolizumab monotherapy as first‐line treatment in NSCLC. In the KEYNOTE‐189 and KEYNOTE‐407 trials, AEs were observed in 99.8% and 98.6% of patients, and irAEs were observed in 26.4% and 35.3%, respectively.^7^, ^8^ In contrast, in the KEYNOTE‐024 and KEYNOTE‐042 trials, AEs were observed in 76.6% and 62.7% of patients, and irAEs were observed in 33.8% and 27.8%, respectively.^5^, ^6^ Although there was no difference in the frequency of irAEs between both therapies, AEs occurred more frequently in patients receiving pembrolizumab plus chemotherapy than pembrolizumab monotherapy in the clinical trials. Although no reports evaluated the safety of pembrolizumab plus chemotherapy in the clinical setting, several reports did evaluate the safety of pembrolizumab monotherapy. Tambo et al.^18^ reported that irAEs occurred in 42.1% of patients treated with first‐line pembrolizumab monotherapy, and Cortellini et al.^22^ reported that irAEs occurred in 32.9% of patients. In the present study, AEs including irAEs occurred more frequently in the combination therapy group than in the monotherapy group and were more frequent than in the clinical trials. Although this result may be due to the extraction of very mild AEs in our study, AEs including irAEs in patients treated with either pembrolizumab plus chemotherapy or pembrolizumab monotherapy may develop more frequently in the clinical setting than in clinical trials. Additionally, nephritis was more frequent in the combination therapy group. Because pembrolizumab was combined with pemetrexed or cisplatin, it was difficult to evaluate whether the side effects were due to pembrolizumab or chemotherapy. In the present study, renal dysfunction was reported collectively as nephritis.

In the present study, treatment discontinuation due to AEs was more frequent in the combination therapy group. In the KEYNOTE‐189 and KEYNOTE‐407 trials, treatment discontinuation due to AEs was observed in 33.6% and 27.3% of patients treated with pembrolizumab plus chemotherapy, respectively,^7^, ^8^ whereas in the KEYNOTE‐024 and KEYNOTE‐042 trials, it was observed in 13.6% and 9.0% of patients treated with pembrolizumab monotherapy, respectively.^5^, ^6^ Several studies showed that irAEs were associated with improvement of prognosis. Haratani et al. reported that among NSCLC patients treated with nivolumab, those with irAEs had significantly longer median OS than those without irAEs (NR vs. 11.1 months).^23^ Ricciuti et al. similarly reported that patients with irAEs had significantly longer median OS than those without irAEs (17.8 vs. 4.0 months), and patients who developed ≥2 irAEs had significantly longer median OS than those with one or no irAEs (26.8 vs. 11.9 vs. 4.0 months, respectively).^24^ However, Ksienski et al. showed that patients with nivolumab or pembrolizumab treatment interruption due to irAEs were associated with a lower median OS than that in the patients with continuous treatment (8.27 vs. 14.54 months).^25^ In the present study, discontinuation due to irAEs until 1 year was more frequent in the combination therapy group than in the monotherapy group. In addition, discontinuation due to AEs other than irAEs occurred and patients who developed multiple AEs of grade ≥3 were more frequent in the combination therapy group. Thus, the occurrence of treatment discontinuation due to AEs was considered high in the combination therapy group, and the addition of chemotherapy to pembrolizumab led to an increase in treatment discontinuation due to AEs, which might be one of the reasons why there was no difference in prognosis between the two groups.

Several reports have evaluated first‐line pembrolizumab plus chemotherapy and pembrolizumab monotherapy for NSCLC patients aged ≥75 years. The phase 3 IMpower150 trial^9^ enrolled non‐squamous NSCLC patients, and the phase 3 IMpower131 trial^11^ enrolled squamous NSCLC patients to evaluate efficacy and safety between first‐line atezolizumab plus chemotherapy and chemotherapy. Patients aged ≥75 years treated with atezolizumab plus chemotherapy comprised 9.0% of patients in the IMpower150 trial and 11.8% of patients in the IMpower131 trial. In the subgroup analysis of patients aged ≥75 years, IMpower150 showed no significant difference in PFS between the two groups, and subgroup analysis was not performed for OS, whereas IMpower131 showed significantly better PFS in patients receiving atezolizumab plus chemotherapy than chemotherapy, but there was no significant difference in OS between them. A pooled analysis of trials evaluating pembrolizumab monotherapy for NSCLC patients showed that patients aged ≥75 years with PD‐L1 TPS ≥50% treated with pembrolizumab as first‐line therapy had longer median OS than those treated with chemotherapy (27.4 vs. 7.7 months).^26^ AEs of grade ≥3 in pembrolizumab monotherapy occurred more frequently in patients aged ≥75 years than in those aged <75 years (24.2% vs. 16.9%). Imai et al. investigated 47 NSCLC patients aged ≥75 years with PD‐L1 TPS ≥50% treated with first‐line pembrolizumab and showed that the ORR was 57.1% and median OS was NR.^12^ They reported that discontinuation due to AEs occurred in 21.3% of patients, which was more frequent than in the clinical trials. The present study revealed that half of the patients obtained a clinical response in both groups. Thus, the clinical response in patients aged ≥75 years may be similar to that in patients aged <75 years, but caution is required due to the potential for increased AEs in patients aged ≥75 years.

In the clinical trials evaluating first‐line pembrolizumab for NSCLC patients,^5^, ^6^, ^7^, ^8^ patients with ECOG PS of ≥2 were excluded. While no reports evaluated first‐line pembrolizumab plus chemotherapy for the NSCLC patients with ECOG PS of ≥2 in the clinical setting, a few reports evaluated first‐line pembrolizumab monotherapy in NSCLC patients. Alessi et al. investigated 234 NSCLC patients with PD‐L1 TPS ≥50% treated with first‐line pembrolizumab, including 195 with ECOG PS of 0 or 1 and 39 with PS of 2, and reported that the patients with PS of 2 had significantly lower ORR and shorter median OS than those with PS of 0 or 1 (25.6% vs. 43.1% and 7.4 vs. 20.3 months, respectively).^13^ Friedlaender et al. also studied 302 NSCLC patients with PD‐L1 TPS ≥50% treated with first‐line pembrolizumab, including 246 with PS of 0 or 1 and 56 with PS of 2, and reported that the patients with PS of 2 had significantly lower ORR and shorter median OS than those with PS of 0 or 1 (45% vs. 72% and 7.8 months vs. NR, respectively).^14^ AEs of grade ≥3 occurred in 9% of the patients with ECOG PS of 2 and in 7% of the patients with ECOG PS of 0 or 1, and there was no significant difference in the incidence of AEs between them. Facchinetti et al. studied 153 NSCLC patients with ECOG PS of 2 and revealed that the ORR was 21%, median OS was 3.0 months, and irAEs occurred in 29% of the patients.^15^ In addition, they showed that patients with ECOG PS of 2 due to comorbidities of the cardiovascular and respiratory systems had longer median OS than those with ECOG PS of 2 due to lung cancer itself (11.8 vs. 2.8 months). Similar to these previous studies, the present study revealed the poor clinical response to pembrolizumab monotherapy in patients with PD‐L1 TPS ≥50% and ECOG PS of 2. However, two patients lived for more than 2 years. Therefore, some patients with poor ECOG PS may benefit from pembrolizumab monotherapy.

This study has several limitations. First, because it was retrospective, some clinical characteristics of the patients were not available. Second, it was performed at a single hospital and only Japanese patients were treated. Third, the sample size was small. Finally, there was a large difference in the observation period between the two groups.

In summary, there was no significant difference in clinical response and prognosis between the combination therapy group and the monotherapy group. In the clinical setting, there were only two patients aged ≥75 years and no patients with poor PS in the combination therapy group. In the monotherapy group, elderly patients may be expected to achieve treatment effects similar to those of younger patients, but the frequency of AEs is higher and requires caution. Additionally, patients with poor ECOG PS may show poorer clinical benefit than patients with good ECOG PS, but some patients may benefit. Although similar to that in the clinical trials, the frequency of AEs was higher in the combination therapy group than in the monotherapy group, but was higher in both groups compared to the clinical trials. We think that the higher frequency of treatment discontinuation due to AEs in the combination therapy group compared tothe monotherapy group is one reason why there was no significant difference in prognosis between the two groups. Prospective trials are needed to evaluate the effects of adding pembrolizumab to chemotherapy in patients with PD‐L1 TPS ≥50%.

## DISCLOSURE

The authors declare no conflicts of interest.

## Supporting information


**Supporting Information Table S1** Adverse events including immune‐related adverse events in patients with PD‐L1 TPS ≥50%
**Supporting information Table S2** Continuation of first‐line treatment in patients with PD‐L1 TPS ≥50%
