# Supplementary material for: A retrospective analysis of pembrolizumab plus chemotherapy versus pembrolizumab monotherapy for advanced or recurrent non‐small cell lung cancer
Source: Thorac Cancer. 2021 Mar 12;12(9):1387–97. doi: 10.1111/1759-7714.13915 (PMC8088931; doi:10.1111/1759-7714.13915)
Supplement: Supplementary file 1 — Supporting Information Table S1 Adverse events including immune‐related adverse events in patients with PD‐L1 TPS ≥50% Supporting information Table S2 Continuation of first‐line treatment in patients with PD‐L1 TPS ≥50% [file TCA-12-1387-s001.docx]

| **Supplementary Table 1. Adverse events including immune-related adverse events in patients with PD-L1 TPS ≥50%** | | | | | | |
| --- | --- | --- | --- | --- | --- | --- |
| Events | **Combination therapy group (n=11)** | | | **Monotherapy group (n=37)** | | |
|  | **All** | **Grade ≥3** | **Discontinuation** | **All** | **Grade ≥3** | **Discontinuation** |
| **Any AEs including irAEs** | 11 (100.0) | 3 (18.2) | 5 (45.5) | 30 (81.1) | 8 (21.6) | 9 (24.3) |
| Anorexia or nausea | 7 (63.6) | 1 (9.1) | 0 (0.0) | 0 (0.0) | NA | NA |
| Neutropenia | 5 (45.5) | 2 (18.2) | 0 (0.0) | 1 (2.7) | 0 (0.0) | 0 (0.0) |
| Anemia | 4 (36.4) | 2 (18.2) | 0 (0.0) | 1 (2.7) | 1 (2.7) | 0 (0.0) |
| Thrombocytopenia | 3 (27.3) | 2 (18.2) | 0 (0.0) | 1 (2.7) | 0 (0.0) | 0 (0.0) |
| Drug-related fever | 1 (9.1) | 0 (0.0) | 0 (0.0) | 0 (0.0) | 0 (0.0) | 0 (0.0) |
| Pneumonia | 1 (9.1) | 0 (0.0) | 0 (0.0) | 3 (8.1) | 1 (2.7) | 0 (0.0) |
| Hiccups | 3 (18.2) | 0 (0.0) | 0 (0.0) | 0 (0.0) | NA | NA |
| Asthma | 0 (0.0) | NA | NA | 1 (2.7) | 1 (2.7) | 0 (0.0) |
| **Any irAEs** | 11 (100.0) | 3 (27.3) | 5 (45.5) | 28 (75.7) | 6 (16.2) | 9 (24.3) |
| Hepatitis | 5 (45.5) | 0 (0.0) | 1 (9.1) | 14 (37.8) | 1 (2.7) | 1 (2.7) |
| Rash | 4 (36.4) | 2 (18.2) | 0 (0.0) | 14 (37.8) | 0 (0.0) | 1 (2.7) |
| Nephritis | 3 (27.3) | 1 (9.1) | 3 (27.3) | 8 (21.6) | 0 (0.0) | 0 (0.0) |
| Colitis or diarrhea | 3 (27.3) | 0 (0.0) | 0 (0.0) | 2 (5.4) | 0 (0.0) | 0 (0.0) |
| Pneumonitis | 1 (9.1) | 0 (0.0) | 1 (9.1) | 7 (18.9) | 3 (8.1) | 6 (16.2) |
| Thyroid dysfunction | 2 (18.2) | 0 (0.0) | 0 (0.0) | 5 (13.5) | 0 (0.0) | 0 (0.0) |
| Isolated ACTH deficiency | 0 (0.0) | NA | NA | 1 (2.7) | 1 (2.7) | 0 (0.0) |
| Arthritis | 0 (0.0) | NA | NA | 1 (2.7) | 0 (0.0) | 0 (0.0) |
| Eosinophilic fasciitis | 0 (0.0) | NA | NA | 1 (2.7) | 1 (2.7) | 1 (2.6) |
| **Total AEs** | 42 | 10 |  | 60 | 9 |  |
| Data are presented as n, median (range) or n (%).  Abbreviations: ACTH = adrenocorticotropic hormone; AEs = adverse events; DVT = deep venous thrombosis; irAEs = immune-related adverse events; NA = not available; PTE = pulmonary thromboembolism. | | | | | | |

| **Supplementary Table 2. Continuation of 1st-line treatment in patients with PD-L1 TPS ≥50%** | | | | | | | | |
| --- | --- | --- | --- | --- | --- | --- | --- | --- |
|  | **End of observation period** | | **At 3 months** | | **At 6 months** | **At 1 year** | |  |
| **Combination therapy group** | | | | | | | | |
| n | | 11 | 11 | 11 | | | 11 | |
| Continuation of 1st-line treatment | | 2 (18.2) | 8 (72.7) | 3 (27.3) | | | 1 (9.1) | |
| Discontinuation due to PD | | 4 (36.4) | 1 (9.1) | 4 (36.4) | | | 4 (36.4) | |
| Discontinuation due to AEs | | 5 (45.5) | 2 (18.2) | 4 (36.4) | | | 5 (45.5) | |
| Discontinuation due to irAEs | | 5 (45.5) | 2 (18.2) | 4 (36.4) | | | 5 (45.5) | |
| Discontinuation due to others | | 0 (0.0) | 0 (0.0) | 0 (0.0) | | | 0 (0.0) | |
| **Monotherapy group** | | | | | | | | |
| n | | 37 | 37 | 37 | | | 37 | |
| Continuation of 1st-line treatment | | 6 (16.2) | 13 (35.1) | 13 (35.2) | | | 7 (18.9) | |
| Discontinuation due to PD | | 21 (56.8) | 18 (48.6) | 19 (51.4) | | | 21 (56.8) | |
| Discontinuation due to AEs | | 9 (24.3) | 6 (16.2) | 6 (16.2) | | | 8 (21.6) | |
| Discontinuation due to irAEs | | 9 (24.3) | 6 (16.2) | 6 (16.2) | | | 8 (21.6) | |
| Discontinuation due to others | | 1 (2.7) | 0 (0.0) | 0 (0.0) | | | 1 (2.7) | |
| Data are presented as n (%).  Abbreviations: AEs = adverse events; irAEs = immune-related adverse events; PD = progressive disease. | | | | | | | | |
